# Quantitative analysis of harmonic convergence in mosquito auditory interactions

**DOI:** 10.1098/rsif.2015.1007

**Published:** 2016-04

**Authors:** Andrew Aldersley, Alan Champneys, Martin Homer, Daniel Robert

**Affiliations:** 1 Bristol Centre for Complexity Sciences, University of Bristol, Bristol BS8 1TR, UK; 2 Department of Engineering Mathematics, University of Bristol, Bristol BS8 1UB, UK; 3 School of Biological Sciences, University of Bristol, Bristol BS8 1TQ, UK

**Keywords:** bioacoustics, acoustic signal processing, hearing, active audition

## Abstract

This article analyses the hearing and behaviour of mosquitoes in the context of inter-individual acoustic interactions. The acoustic interactions of tethered live pairs of *Aedes aegypti* mosquitoes, from same and opposite sex mosquitoes of the species, are recorded on independent and unique audio channels, together with the response of tethered individual mosquitoes to playbacks of pre-recorded flight tones of lone or paired individuals. A time-dependent representation of each mosquito's non-stationary wing beat frequency signature is constructed, based on Hilbert spectral analysis. A range of algorithmic tools is developed to automatically analyse these data, and used to perform a robust quantitative identification of the ‘harmonic convergence’ phenomenon. The results suggest that harmonic convergence is an active phenomenon, which does not occur by chance. It occurs for live pairs, as well as for lone individuals responding to playback recordings, whether from the same or opposite sex. Male–female behaviour is dominated by frequency convergence at a wider range of harmonic combinations than previously reported, and requires participation from both partners in the duet. New evidence is found to show that male–male interactions are more varied than strict frequency avoidance. Rather, they can be divided into two groups: convergent pairs, typified by tightly bound wing beat frequencies, and divergent pairs, that remain widely spaced in the frequency domain. Overall, the results reveal that mosquito acoustic interaction is a delicate and intricate time-dependent active process that involves both individuals, takes place at many different frequencies, and which merits further enquiry.

## 1. Introduction

Collective motion in groups of mosquitoes and other swarming dipterans has been the subject of much recent study [1–6], as it has for many other animals [7–11]. At the heart of these investigations is a fundamental question: what is the nature of the interactions between individuals within the group that produce such emergent behaviours? This paper aims to explore this question from the perspective of measurement and analysis of the interactions; specifically, we investigate the auditory interactions between pairs of mosquitoes.

Audition has long been known to be central to mosquito interaction. Male mosquitoes of many species form swarms that attract females, who approach individually to locate a mate [12–19]. Being part of a swarm increases the likelihood of any individual male encountering a female [20]. Once a potential mate is located, males orient themselves according to flight tones produced by females [21–25]. The male's hearing apparatus is tuned to actively and nonlinearly respond to the tonal signal of a passing female, amplifying her weak sound and thus enhancing his ability to track and pursue her [26–28]. As well as performing the tasks of detection and positional resolution, mosquitoes may also use the characteristic sounds of their neighbours to inform mate choice and infer gender and/or species identification [29–33].

At a pairwise level, mosquitoes participate in an animated frequency-based interaction, known as ‘harmonic convergence’ [30]. This was first reported by Gibson & Russell [29], who observed an increase in the variability of wing beat frequencies of pairs of males and females, of the species *Toxorhynchites brevipalpis*, when compared with solo flight. Significantly, opposite sex pairs of these mosquitoes displayed an apparent effort to converge their wing beats to a common frequency value. Subsequent studies have demonstrated similar flight frequency matching behaviours in other species of mosquito: *Culex quinquefasciatus* [31], *Aedes aegypti* [30] and *Anopheles gambiae* [33].

Mosquito wings have numerous vibrational modes, producing acoustic signals that comprise a high-energy fundamental frequency and multiple, successively weaker, harmonic overtones [34,35]. The wing beat frequencies of male and female *T. brevipalpis* occupy broadly similar ranges, and thus frequency-based interactions take place at the fundamental flight frequency, yet this is not the case for other species, which display a greater degree of sexual dimorphism. In such mosquitoes, convergence of wing beat frequencies has been observed to take place at the lowest shared harmonic overtone, typically the male second and female third flight frequency component [30,31].

While a great deal of progress has been made in our understanding of acoustic signalling and processing in mosquitoes, a quantitative description of their flight frequency behaviours remains lacking. This is particularly true of ‘harmonic convergence’, which has no formal definition, and for which several interpretations have been given in the literature (e.g. [31,33,36,37]). Moreover, while it has been stated that ‘there is little doubt that “frequency convergence” occurs’ [38], it has not yet been quantitatively demonstrated that it is indeed an active behaviour, and not simply a random, passive, occurrence that results by chance, because insects occupy a common band of frequency space. For example, the male *A. aegypti* has a fundamental wing beat frequency approximately 50% higher than that of the female. When flying in a pair, the individuals may therefore be expected to have a natural overlap around the region of the male's second and female's third harmonic components, without any active flight frequency modulation being necessary.

Previous studies have typically visualized mosquito flight tones using a spectrogram representation of wing beat frequency [29–31,33,36,37]. Such Fourier-based approaches demand a trade-off between resolution in the time and frequency domains, and are inherently difficult to extract accurate data from [34]; it is therefore quite possible that there exist aspects of mosquito frequency interactions which may not be apparent when analysed in this way.

In this study, we seek to address these concerns, and consolidate and expand upon existing knowledge of mosquito bioacoustics by providing a thorough quantitative evaluation of high-quality audio recordings of tethered live mosquito pairs, as well as of tethered individuals subjected to playback recordings. We apply Hilbert spectral analysis [34] to calculate instantaneous frequency time series, which yields acoustic interaction data at a higher time–frequency resolution than has previously been reported. This more accurate analysis generates data that in turn motivate the development of tools enabling the quantitative characterization of mosquito acoustic behaviours. In particular, we investigate how the acoustic interactions of male–male pairs—of which investigations are sparse—compare to those of opposite sex pairs. We also test, quantify, and expand on the phenomena reported in [30], where both male and female mosquitoes were found to harmonically converge to a playback stimulus (i.e. a non-interactive sound signal) of the opposite sex. We aim to provide robust statistical evidence to determine whether harmonic convergence between mosquito pairs is indeed a genuine phenomenon and, if so, identify the different ways it can be observed (in both the frequency and time domains), quantify how likely it is to occur, and how behaviour differs across a population.

Here, we describe a series of experiments designed to investigate pairwise interactions between opposite and same sex mosquitoes. We introduce analytical tools developed to probe communication in the frequency domain, with a particular emphasis on the reliable detection of unique convergence events. Finally, we discuss the implications of our findings in the context of new experimental approaches to characterize swarm dynamics and mosquito mating behaviour.

## 2. Experimental methods

### 2.1. Mosquito husbandry

A colony of the species *A. aegypti* was initiated using laboratory eggs obtained from the London School of Hygiene and Tropical Medicine. Mosquitoes were developed in containers filled with de-oxygenated water in a humidity controlled environment, held at approximately 26°C. Pupae were routinely extracted into vials, which were then placed open in secured flight cages, running on a D16 : N8 circadian cycle. Emerged adults were fed a diet of 10–20% glucose solution.

### 2.2. Preparation and test conditions

We recorded flight tones of live mosquitoes less than one-week old following the procedure outlined in [34]. Test subjects were anaesthetized for 10–20 s using a stream of carbon dioxide gas and tethered to the rounded end of an entomological stainless steel pin (gauge 000). After allowing sufficient time for recovery (around 15 min), mosquitoes were introduced to the experimental arena. Insects were positioned upright in such a way as to mimic their natural flight positions. Flight was initiated either by gently blowing on each mosquito or by stroking its legs. Acoustic emissions were recorded using two electret condenser microphones (FG-23329-C05, Knowles Electronics), one placed directly underneath each animal. The acoustic sampling frequency was set at 40 kHz. Flight tones were recorded on separate channels, logged simultaneously using a custom-built integrating amplifier (based on a published circuit design [39]), prior to being passed into a National Instruments (National Instruments, Austin, TX, USA) USB data acquisition device. Data were logged digitally using LabVIEW SignalExpress (National Instruments).

Temperature, humidity and lighting conditions were monitored and controlled throughout experimental trials. Recordings were carried out in a soundproofed booth to reduce noise effects from external sources. Care was taken to minimize disturbance of the mosquitoes' visual field by pointing them towards the rear of the darkened enclosure.

Note that the effect of tethering on the biomechanics of mosquito flight is uncertain [40], despite its widespread use in studies involving insect flight kinematics [29–31,33,36,37,41]. While the pin itself does not create resonance artefacts (see, e.g. supplementary material of [30]), there is evidence that tethering results in a decrease in mosquito wing beat frequency compared with free flight [30,35,42]. Furthermore, there may be differences in frequency composition of the flight tone between hovering and forward flight, and the variation of wing beat phase around the mosquito implies that orientation in free flight (fixed in our tethered experiments) may also be important [35]. While an analysis of mosquito communication in natural conditions is clearly a major goal, the number of variables in free flight and the challenging nature of the experiments motivate the simpler tethered experiments we undertake here, in the belief that at least some aspects of free flight behaviour are preserved in tethered individuals.

### 2.3. Experimental procedure

Our experiments had two areas of focus. First, we studied acoustically mediated behavioural interactions between live pairs of same and opposite sex mosquitoes. Next, we investigated the response of individual mosquitoes to playbacks of pre-recorded flight tones. The methods employed for each scenario are detailed below.

#### 2.3.1. Live pairwise recordings

**Figure 1.**
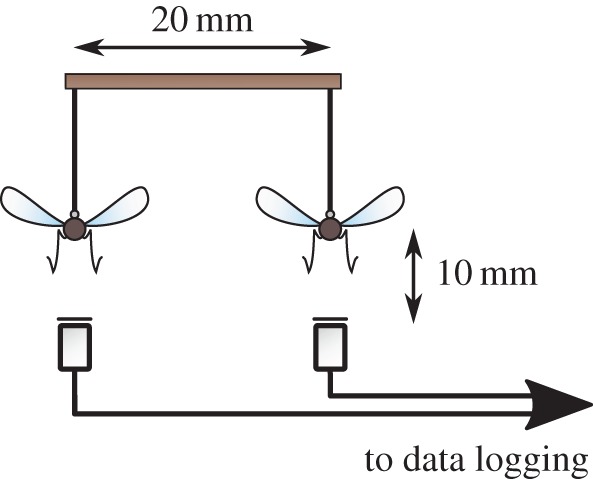
Schematic of experimental set-up for live pairwise recordings; flight tones of each mosquito are recorded on separate channels via microphones placed directly below each insect.

Opposite and same sex pairs of mosquitoes were positioned next to each other at a distance of around 20 mm; figure 1 shows a schematic of the experimental set-up. This is well within their range of acoustic detection [27], but beyond the reach of physical contact. After stimulating flight in both individuals, the sounds produced by each were recorded for a duration of 60 s.

Between experiments, mosquitoes were kept in isolated containers to minimize extra-recording acoustic interaction. In some instances, multiple recordings were taken from the same pair; a minimum time of 10 min was left between such repeat trials.

In these tests, data were recorded from each subject on an independent audio channel [34], in contrast to single-microphone techniques used in all previous studies (e.g. [29–31,33,35]). Significantly, our protocol [34], based on the Hilbert transform, enables rapid and accurate quantification of the wing beat frequency of the mosquitoes.

#### 2.3.2. Playback recordings

Live male and female mosquitoes were subjected to a mixture of pre-recorded flight tones of lone and paired conspecifics, drawn at random from an archive developed earlier by Aldersley *et al*. [34]. Tethered individuals were placed 40 mm in front of and facing a loudspeaker, through which playback tones were fed at a sound pressure level of 86 dB, which approximates the intensity of a female mosquito acoustic emissions measured at 20 mm [27]. Mosquitoes were recorded for 5 s prior to the introduction of the playback stimulus, which lasted for 60 s, followed by a further 5 s of lone, stimulus free, flight.

## 3. Development of analytical tools

### 3.1. Data preprocessing and frequency extraction

The study of behavioural interactions relies fundamentally on a detailed spectral and temporal representation of each mosquito's flight sequences. We apply the concept of instantaneous frequency, obtained via Hilbert spectral analysis [43], to our collection of audio recordings. The key advantage of Hilbert spectral analysis is that it yields a time series of instantaneous wing beat frequency, with the same sampling rate as the original data. Thus, it achieves a significant improvement over other techniques in both the time and frequency resolution; in particular, short-time fast Fourier transforms are fundamentally limited by an inherent time–frequency trade-off and are unable to capture the rapid modulations in frequency exhibited by mosquito flight tones.

Data were processed according to the methodology detailed in [34], briefly summarized as follows. To isolate the fundamental frequency component, a time-varying filter is applied to the raw acoustic waveform associated with a given mosquito. The instantaneous frequency, *ω_i_*(*t*), can then be determined via Hilbert transformation. Higher harmonic frequencies are obtained by integer multiplication of the fundamental; such a linear relationship between overtones has been shown to hold even during periods of frequency modulation [35], and is also valid in the data presented here.

This approach yields mosquito acoustic interaction data with a time and frequency resolution at the sampling rate used during data acquisition (40 kHz). An automated process is then used to detect and remove portions of the recordings during which mosquitoes temporarily cease flight [34], the absence of a flight tone in the signal being detrimental to quantitative analysis.

### 3.2. Detecting frequency convergence

In *Aedes aegpyti* mosquitoes, harmonic convergence has been reported to take place most commonly at the male second and female third frequency overtone, and to a lesser degree at the male first and female second overtone [30]. Male–male pairs of mosquitoes, on the other hand, interact at the level of the fundamental frequency component. In general, interactions in the frequency domain occur over some range that is shared by the mosquitoes in the pair, and are apparent at integer ratios between their fundamental flight frequencies.

Here, we take an approach similar to that of [31,33], in which fundamental wing beat frequency ratios between mosquitoes were used to explore convergent relationships between mosquitoes. Constant flight frequency ratios indicate a fixed frequency relationship between the mosquitoes; when located at rational fractions (with small integer numerator and denominator), this points towards harmonic convergence. For pairs of signals *ω_i_*(*t*),*ω_j_*(*t*), we calculate the instantaneous ratio between them, using the lower frequency as the denominator. The ratio is uniquely determined because the signals *ω_i_*(*t*) are of the fundamental frequency component. The resultant time series serves a number of functions. Firstly, by looking at the distribution of frequency ratios at the population level, we are able to identify common occurrences in the frequency behaviours of our experimental cohort. Secondly, ratios can be used to consistently assign convergence events within individual pairs. Both of these ideas are developed further below.

#### 3.2.1. The randomization protocol

A key aim of this study is to establish frequency convergence as an active or passive phenomenon, i.e. do mosquitoes make an effort to converge at harmonic frequencies, or does this observation simply result from chance? One way to answer this question is to compare the distribution of frequency ratios between opposite and same sex pairs to those of ‘non-interactive’ pairs.

Consider pairing the recording of a male mosquito in lone flight with that of a female also flying solo (data contained in the ♂ and ♀ groups, respectively, as summarized in table 1). These mosquitoes are in no way interacting with one another, and should indeed be beating their wings in accordance with their ‘rest’ state, typified by a flight frequency distributed approximately normally [34]. However, by combining their wing beat frequency traces, we create a dataset which can be subjected to the same analysis as that generated by a live pair (the ♂♀ dataset). Apparent convergence in such pairs can be considered truly random, since there is no way for these mosquitoes to have modulated their flight frequencies in response to a neighbour.

**Table 1.** Experimental summary statistics for male (♂) and female (♀) mosquito populations and combinations. Subscript ‘P’ denotes a playback recording; for example, ♂♀_P_ indicates a recording of a live male mosquito subjected to a female playback. Columns 2–3 give the mean wing beat frequency, along with its minimum and maximum range. Standard deviations are reported for both within (*σ*_intra_) and between (*σ*_inter_) different recordings. We also show another measure of flight frequency dispersion: the inter-quartile range, for which we report the mean within-record value (IQR_intra_), and the range over all individuals in each pair type.

		*μ* (range) (Hz)		*σ*_intra_ (Hz)		*σ*_inter_(Hz)		IQR_intra_ (Hz)	
type	*N*	♂	♀	♂	♀	♂	♀	♂	♀
♂	27	691.2 (492.1–880.2)	—	11.1	—	90.5	—	15.1 (5.8–33.4)	—
♀	19	—	479.8 (415.9–527.0)	—	9.7	—	31.3	—	12.8 (2.9–37.9)
♂♀	43	682.5 (493.3–881.8)	456.7 (350.8–562.7)	12.9	8.9	101.4	42.3	18.2 (3.7–63.8)	12.2 (3.7–40.6)
♂♂	30	684.4 (568.5–799.3)	—	14.8	—	60.2	—	20.1 (3.9–54.8)	—
♂♀_P_	34	628.9 (524.9–745.0)	—	9.6	—	55.8	—	13.3 (2.8–44.3)	—
♀♂_P_	34	—	468.5 (352.5–550.9)	—	12.6	—	50.7	—	18.8 (5.5–61.6)

Permuting across lone recordings of male and female mosquitoes in this manner supplies a population of ‘random’ pairs (*N* = 68) that can be used to benchmark convergence between the interactive pairs that we have recorded, by comparing the prevalence of that behaviour between the ‘real’ and ‘artificial’ pairs. We are not aware of the use of any such re-sampling techniques in the mosquito bioacoustics literature to date.

### 3.3. Classifying individual convergence events

For pairwise mosquito interactions, frequency convergence events must be quantified in a robust and unbiased manner, a need that was highlighted by previous studies [31,33,36,37]. A framework is developed here to consistently detect frequency convergence in mosquitoes, with the aim of facilitating more straightforward comparisons between future acoustic studies in various species.

#### 3.3.1. The piecewise aggregate approximation

One of the most fundamental challenges in any investigation involving time-series data is to identify features and similarities across the database of interest. This is particularly true for high-resolution, high-dimensionality, noisy data, categorizations, which can certainly be applied to the time series presented here. Consequently, there has been much interest in the development of data-mining algorithms that can be used to classify whole time series, or subsets therein, according to some particular criteria. The piecewise aggregate approximation (PAA) [44,45] offers a simple yet powerful means to reduce the dimensionality of a time series, which can aid us greatly in prescribing wing beat frequency convergence between mosquitoes. An outline of the PAA now follows.

Consider a time series *X* of length *n* as a set of discrete points such that *X* = *x*_1_, …, *x_n_*, that we wish to reduce to a representation of dimension *N*, where 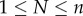 and, typically, 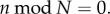 The reduced form of *X* in *N*-dimensional space is denoted 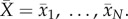 Using PAA, the *i*th element of 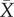 is then determined as3.1
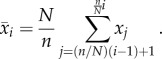


**Figure 2.**
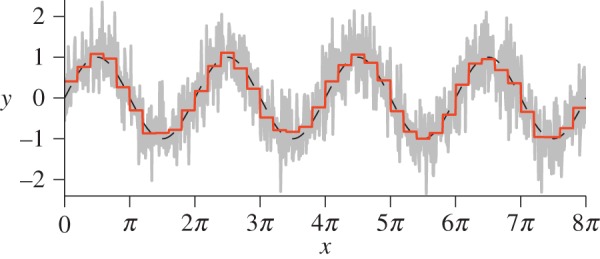
A demonstration of how PAA can be used to reduce the dimensionality of a time series. A sine wave with additive Gaussian noise (grey) is decomposed into short-time segments, for which the mean value is calculated. The reconstructed representation (red solid line) approximates the ‘true’ signal (black dashed line) well.

In essence, a given time series is divided into *N* equally sized frames, and the mean value of each is calculated. A vector of the combined means becomes the reduced representation of the original data (an illustrative example is given in figure 2).

#### 3.3.2. Applying piecewise aggregate approximation to frequency-based mosquito interactions

**Figure 3.**
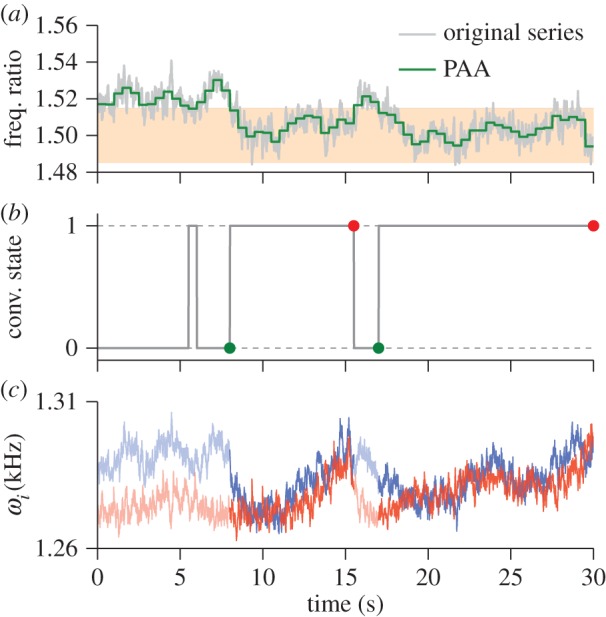
Using PAA to identify harmonic convergence in mosquito pairs. (*a*) Determine the ratio of fundamental flight frequencies between the paired mosquitoes, and take the PAA using a window of size *w* = 0.5 s. Shaded region indicates convergence tolerance *δ* = ±1% about the integer ratio 3 : 2 (1.5). (*b*) Convergent behaviour is assigned a binary state value according to whether the frequency ratio PAA lies on the interval *r* ± *δ*%, here [1.485, 1.515]. A value of 1 indicates convergence. The start and end indices of each convergence ‘event’ (green and red markers, respectively) are then logged, using criteria *τ* = 1 s Δ*τ* = 1 s (i.e. each event must be at least 1 s long and distinct events are separated by more than 1 s). (*c*) Visualization of the prescribed convergence events in the time–frequency plane, at the male (blue) and female (red) second and third harmonic overtones, respectively.

As described earlier, harmonic convergence is characterized by the maintenance of a fixed integer ratio between the fundamental frequencies of two mosquitoes in a pair. This process, however, is inherently affected by noise, a fact that becomes obvious when analysing the highly temporally-resolved data that we have obtained here (an example is shown in figure 3). Hence, it can be difficult to assess where convergence begins and ends and how long individual events are. Reducing the dimensionality of our data via PAA (applied to the instantaneous frequency data obtained from Hilbert spectral analysis) helps simplify this process. While being computationally inexpensive and easy to implement, PAA rivals other more sophisticated dimensionality reduction techniques (such as wavelet and Fourier decompositions) [44,45], and comfortably satisfies the requirements for this classification task. Thus, it has the twin benefits of reducing the computational cost of our study, and of improving the robustness of the detection of harmonic convergence to noise.

It is important to bear in mind that identifying convergence events necessarily requires certain restrictions to be imposed upon the data. We determine the PAA representation of the ratio of fundamental wing beat frequencies for the pair using a window of size *w*. Convergence events are labelled as contiguous time intervals of duration *t*, greater than some threshold *τ*, for which the reduced ratio is within a given tolerance *δ* of some integer fraction. Unique events are separated by a time period Δ*τ*. As convergence is rarely perfect, the tolerance parameters *t* and *δ* are essential to the classification process. Here, we use parameters *w* = 0.5 s, *δ* = ±1%, *τ* = 1 s and Δ*τ* = 1 s, which robustly detect convergence events.

A graphical overview of how PAA is used in the context of detecting harmonic convergence events between pairs of mosquitoes is provided in figure 3. Using this routine, we are able to perform an automated and quantitative search for harmonic convergence in our various combinations of mosquito pairs. Once such convergent intervals have been identified, the full-resolution frequency data (i.e. without PAA) are used for further analysis.

## 4. Results

### 4.1. Summary statistics

The numbers and different types of paired recordings obtained during the investigation are presented in table 1, along with statistics for lone male (♂) and lone female (♀) mosquito populations. As in previous studies [30,35,46], tethered male *A. aegypti* consistently flew at a higher fundamental frequency than females (691.2 versus 479.8 Hz for lone insects, and 673.5 versus 465.5 Hz across all recordings, Mann–Whitney *U*-test: 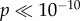), and also displayed greater spread between their average fundamental flight frequencies (s.d. 90.5 versus 31.3 Hz, Levene's test: *p* < 10^−5^). While the male population tends to occupy a broader section of frequency space, variation between individuals (*σ*_inter_) was significantly greater than that within any given recording of an individual (*σ*_intra_).

### 4.2. Convergence in opposite sex pairs

The analytical techniques outlined in §3 allow us to go far beyond this summary analysis, and to analyse the data to provide quantitative measures of harmonic convergence. This permits a much more detailed investigation of frequency interactions at both individual and population level than has hitherto been possible. We begin by analysing acoustic data collected from live (♂♀) and all playback (♂♀_P_ and ♀♂_P_) opposite sex pairs of *A. aegypti*.

#### 4.2.1. Time–frequency representation

**Figure 4.**
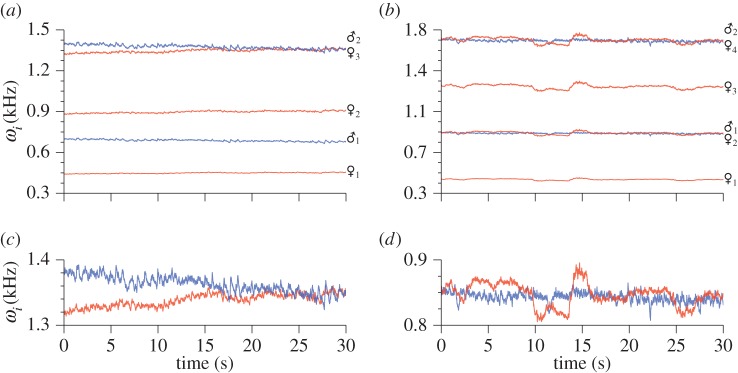
Examples of harmonic convergence, viewed in the form of a frequency time plot. Convergence is typified by one or both of the mosquitoes altering their wing beat frequency so that harmonic components approximately converge to some low integer ratio, for a period lasting up to tens of seconds. The relevant frequency components are shown with zoomed-in frequency axis (over the same time) below. The data presented here are extracted using Hilbert spectral analysis, and show convergence at female : male integer ratios of (*a*) 3 : 2 and (*b*) 2 : 1. At this resolution the imperfect nature of frequency convergence between the insects is apparent.

Figure 4 presents examples of instantaneous frequency data (for the fundamental and higher harmonics) as a function of time, for mosquitoes in two sample recordings from the ♂♀ cohort. In each instance, the phenomenon of ‘harmonic convergence’ is visible, as is the possibility that it is mediated through frequency modulation by both sexes during the interaction. It is clear, at this resolution, that an exact match of harmonic components is rarely achieved. Figure 4 also shows that convergence can take place at multiple harmonic combinations even in the same species; in this case, at the female third and male second harmonic (figure 4*a*), and female second harmonic and male fundamental frequency (figure 4*b*), an observation that is consistent with previously reported findings for *A. aegypti* (e.g. [30]).

**Figure 5.**
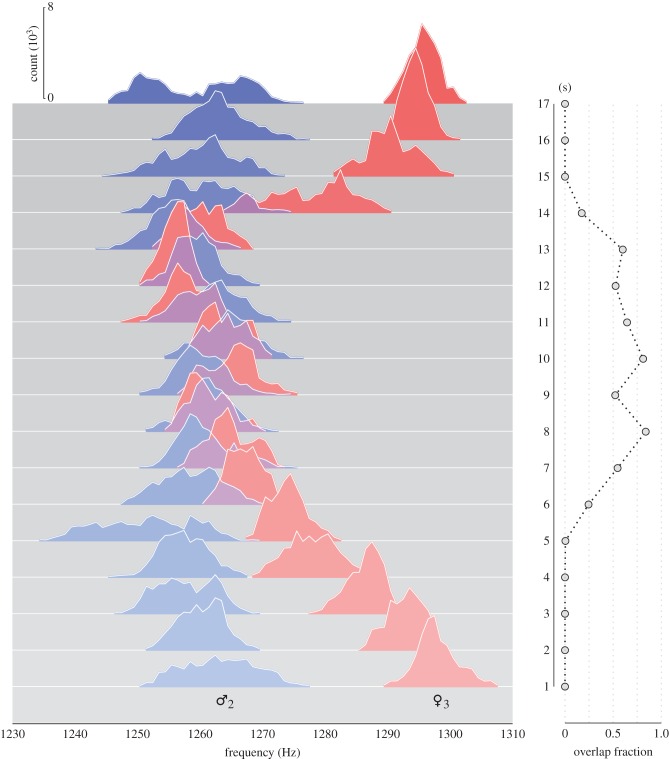
Waterfall plot showing the evolution of wing beat frequency distributions (left) of male (second harmonic, blue) and female (third harmonic, red) mosquitoes, and overlap fraction (right), as functions of time, for a live male–female pair (taken from dataset ♂♀) during a period of harmonic convergence. Distributions and overlaps are calculated using a window size of 1 s at a frequency resolution of 1 Hz.

An alternative perspective on the harmonic convergence phenomenon, permitted by our high-quality data, is shown in figure 5. Here, data at convergent harmonic overtones are aggregated over short time intervals to produce snapshot distributions of wing beat frequency, plotted in a ‘waterfall’ fashion against time. Steady flight is characterized by a sharp-peaked frequency distribution, whereas transient or erratic modulations of flight frequency tend to produce a broader shape. Harmonic convergence, then, can be typified by a gradually increasing overlap between the frequency distributions of the pair (as can be seen in figure 5), akin to ‘shared information’ between the mosquitoes. This overlap, *α*, between distributions *a* and *b*, is quantifiable according to the following metric:4.1
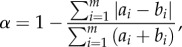
where *m* is the number of bins used to generate frequency histograms. Frequency overlap is plotted as a function of time adjacent to the distributions (figure 5). Interestingly, convergence is markedly imperfect in the overlap measure, with distribution overlaps as low as 30–40%, with a maximum in this case of around 80%.

#### 4.2.2. Convergent behaviours across the population

In each of the datasets presented in figure 4, one would expect that an inspection of the distributions of the female : male flight frequency ratios would show peaks around values of 1.5 and 2 (respectively, for figure 4*a*,*b*). But what can be said of the rest of our experimental data?

In general, the use of frequency ratios is an efficient means to examine convergence behaviours in mosquito pairs, as it reduces the scope of the time–frequency search space, and condenses the information from all harmonic overtones into a single one-dimensional time series. Moreover, it allows us to aggregate and compare data from across the experimental population, as it gives a standardized representation of the acoustic interaction field that is independent of the exact wing beat frequencies displayed by the individuals in the pair. Finally, it does not require any prior knowledge as to the ratios at which convergence may or may not occur. *Aedes aegypti* mosquitoes can detect sounds at frequencies up to around the fifth male harmonic—a range that extends to several thousand Hertz—using difference tones corresponding to the separation between the mosquito's own wing beat and that of its partner [47].

**Figure 6.**
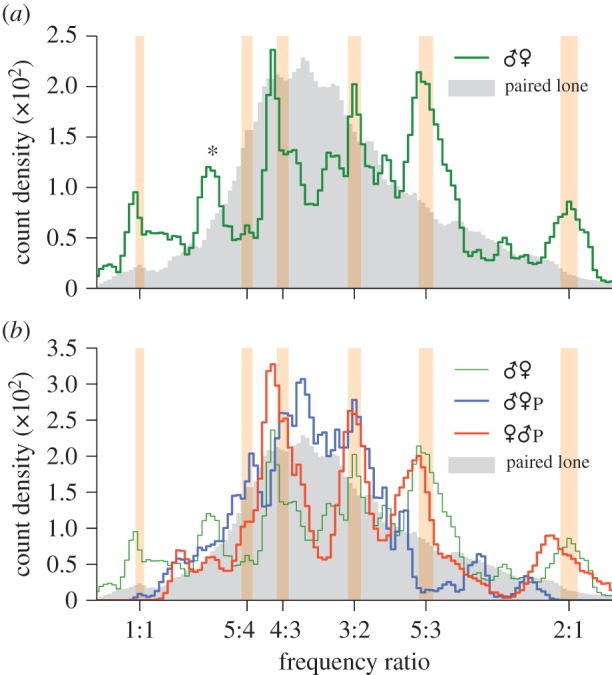
Distribution of fundamental flight tone ratios between fundamental frequencies for (*a*) all *live* paired male–female mosquitoes and (*b*) individual males (blue) and females (red) subjected to playback sounds of the opposite sex. Common harmonic ratios (±1%) are indicated by shaded vertical bars. In each graph, shaded grey area indicates the distribution of fundamental frequency ratios for random combinations of recordings of opposite-sex solo mosquitoes, i.e. those that are not interacting with one another. The live paired data from (*a*) are also presented in (*b*), for comparison.

Figure 6*a* shows the distribution of frequency ratios for all live male–female pairs recorded (♂♀ dataset, *n* = 43), together with that of ‘artificial’ pairs generated by combining recordings of males (♂ dataset, *n* = 27) and females (♀ dataset, *n* = 19) in solo flight. If convergence were a chance phenomenon, one would anticipate that the shapes of these distributions would be broadly similar. However, the shapes of the distributions are markedly different: there are numerous peaks at integer ratios between the actual pairs that do not appear in the artificially paired mosquito data. This is strong evidence that frequency convergence via wing beat modulation is indeed an active process carried out by mosquitoes. Not only this, it suggests that frequency convergence is a two-way phenomenon that requires feedback from both participants in the duet; that is, a mosquito modulates its wingbeats differently when a partner also modulates theirs. However, it is also hypothetically possible that a physical interaction between local airborne pressure variations driven by and influencing the mechanical beating of the wings is responsible for harmonic convergence, at least in part. We cannot, based on our data, rule out this hypothesis, which we discuss further in §5.

Our methodology enables the identification of different modes of convergence between male and female *A. aegypti* (figure 6*a*). While interactions at female : male frequency ratios of 3 : 2 and 2 : 1 have been observed before in this species [30,37], our analysis indicates that harmonic convergence may also take place at fundamental frequency ratios of 1 : 1, 4 : 3, 5 : 3 and 5 : 4. Note that we have excluded harmonic frequencies above the male fourth and female fifth due to the low energy content in the acoustic signal [35] and the low sensitivity of the mosquito hearing organ beyond this range [47]. Therefore, the rather prominent peak at the ratio 7 : 6 (* in figure 6*a*) is not included in our analysis of convergence.

#### 4.2.3. Playback recordings

Figure 6*b* shows the frequency ratio distribution obtained when individual tethered live male or female mosquitoes were subjected to playback recordings of the opposite sex (♀♂_P_ and ♂♀_P_ datasets, respectively, *n* = 34 in both cases). Both sexes displayed the ability to converge to the playback tones, as evidenced by the occurrence of numerous peaks at integer ratios.

Males exposed to playbacks of females show prominent convergent relationships at frequency ratios of 5 : 4, 4 : 3 and 3 : 2, whereas for females stimulated by the sounds of males peaks appear at 4 : 3, 3 : 2, 5 : 3 and, arguably, 2 : 1 (figure 6*b*). The distributions of frequency ratios for both the ♂♀_P_ and ♀♂_P_ datasets differ from those of randomly associated lone pairs (although the ♂♀_P_ to a lesser degree), and peaks occur, on the whole, at the same locations as the live ♂♀ pairs (figure 6*a*), with the exception of 1 : 1. One immediate implication of these findings is that both male and female *A. aegypti* are able to converge their flight tones to playback sounds of the opposite sex. On the other hand, neither of the playback distributions closely matches that of the live pairs, indicating differences in behaviour between unidirectional and bidirectional opposite-sex auditory interactions.

### 4.3. Wing beat frequency interactions in male–male pairs

**Figure 7.**
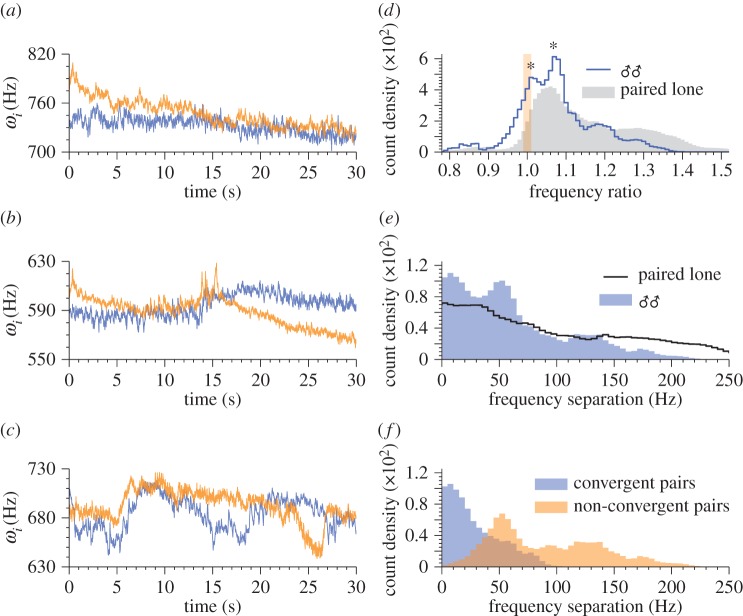
(*a*–*c*) Sample time–frequency spectra of male–male pairs, showing the fundamental flight component only. A variety of behaviours is observed, including apparent sustained convergence qualitatively identical to that of male–female duets (*a*), brief convergence, ‘hunting’ [29], and divergence (*b*), and prolonged frequency modulation (*c*). (*d*) The distribution of ratios between fundamental frequencies for paired males (solid line), plotted along with the equivalent distribution for combinations of males in solo flight (shaded). Pair ratios peak at non-integer values (1.02, 1.09, *): the bimodal peak is not present in the ratio distribution for ‘non-interactive’ pairs. (*e*) The distribution of absolute frequency separations between fundamental frequency components for paired males (shaded), plotted along with the equivalent distribution for combinations of males in solo flight (solid line). Peaks occur in the region of 0–20 and 40–60 Hz, suggestive of a dual-state interaction. (*f*) Splitting the male–male population into convergent and non-convergent pairs largely accounts for the bimodality observed in (*e*).

Mosquito flight frequency convergence has been studied primarily as a precursor to male–female copulation [33–37], and as a result the behavioural interactions of male–male pairs have received notably less attention. Despite this, frequency convergence behaviours that are qualitatively analogous to those of opposite sex pairs have been observed between males [29], and indeed are also present within our data (one example is shown in figure 7*a*); this would also be consistent with the physical interaction hypothesis. For same sex pairs, convergence events may be characterized by frequency ‘hunting’ [29], typified by rapid frequency modulations performed by one or both males when in close frequency proximity to a partner, followed by frequency divergence (figure 7*b*). This has led to the conclusion that pairs of male mosquitoes do not perform frequency convergence, and tend to avoid each other acoustically [31,32]. Here, we see a more dynamic picture, that can include both frequency convergence and avoidance behaviour (figure 7*c*).

Through inspection of the distribution of fundamental wing beat frequency ratios for male–male pairs (♂♂ dataset, *n* = 30, figure 7*d*), we find two peaks—at 1.02 and 1.09—neither of which are at integer ratios. Note that in [31], pairs of male *C. quinquefasciatus* displayed prominent frequency ratios at similar values of 1.07 and 1.13. Comparing these recordings to artificial pairs generated by combining lone individuals (from the ♂ dataset), we find that neither peak from the frequency ratios of the actual pairs is present in that of the non-interactive pairs (figure 7*d*). Indeed, the bimodality of the distribution of ratios in our experimental data, coupled with the equivalently dual-peaked nature of male pairs in [31], suggests that there may be some active significance to these values when transformed into frequency space. Moreover, there is a significant component in the distribution of actual pairs around the integer ratio of 1 : 1, indicating that live male pairs spend a greater amount of time in a state akin to frequency convergence than one would expect if such interactions were ‘random’.

Acoustic interactions between male mosquitoes take place about the fundamental frequency component, and not at higher harmonics as for opposite sex pairs. It is therefore useful to consider how the ratios identified (figure 7*d*) manifest themselves in frequency space. Figure 7*e* shows the distribution of absolute instantaneous separations, in the frequency domain, between each pair of males in the experimental population. Again the distribution is bimodal, suggesting that, when paired with a member of the same sex, males tend to exist in one of two states in terms of their frequency separation: 0–20 Hz (near-convergence), or 40–60 Hz (fixed divergence). Once again, these modes of behaviour are not apparent in the artificially created pairs. When the cohort is split into two groups—those in which at least one convergence event is identified (using the algorithm described in §3.3), and those in which no convergence is observed—we are able to account for the bimodality in the frequency separation distribution (figure 7*f*). Males that converge spend much of their time at a wing beat frequency separation less than 25 Hz apart, whereas the separation for pairs that do not converge tends to be greater than or equal to 50 Hz.

### 4.4. Prevalence of convergence within the population

The analysis presented above strongly suggests that harmonic convergence between pairs of mosquitoes is a genuine phenomenon performed by both sexes, and furthermore that convergence is possible at several different frequency ratios. However, the question still remains of just how likely harmonic convergence is to be observed, either in the time-domain for a given pair of mosquitoes, or at all across a population. To answer this, we use the algorithms described in §3 to consistently capture individual harmonic convergence events within our experimental data, and compute statistics across them.

We begin by conducting a systematic and automated search for convergence among all mosquito pairs at the harmonic integer ratios identified above (1 : 1, 5 : 4, 4 : 3, 3 : 2, 5 : 3 or 2 : 1 for female : male, and 1 : 1 for male : male). A summary of the numbers of experimentally observed convergence events is presented in table 2. We see that convergence is ubiquitous for both male and female mosquitoes, whether they are in live pairs or subjected to a playback recording, of either the same or opposite sex.

**Table 2.** Summary statistics of convergence events in mosquito pairs: live male–male (♂♂) and male–female (♂♀), live female–playback male (♀♂_P_), live male–playback female (♂♀_P_) and all opposite sex live–playback (♂♀_[P]_) experiments. Columns 2–5 represent the total number of recordings, the number (and proportion of the total number) of recordings with at least one convergence event, together with number (and proportion of the total number) of multiple convergence events, and convergence events at multiple frequency ratios. Columns 6–8 represent similar data for unique pairs. Columns 9–11 reports the total, per recording and per unique pair counts of convergence events across all recordings.

	recordings				unique pairs			convergence event count		
		convergence event count				convergence event count				
type	total	at least 1	more than 1	multiple ratio	total	at least 1	multiple ratio	total	per recording	per unique pair
♂♂	30	14 (47%)	10 (33%)	—	18	10 (56%)	—	39	1.3	2.2
♂♀	43	37 (86%)	26 (60%)	4 (9%)	24	22 (92%)	7 (16%)	111	2.6	4.6
♂♀_P_	34	26 (76%)	19 (56%)	5 (15%)	7	7 (100%)	6 (86%)	96	2.8	14
♀♂_P_	34	33 (97%)	26 (76%)	5 (15%)	7	7 (100%)	5 (71%)	126	3.7	18
♂♀_[P]_	68	59 (86%)	45 (66%)	10 (15%)	14	14 (100%)	11 (79%)	222	3.3	16

### 4.5. Convergence duration and harmonic combinations

**Figure 8.**
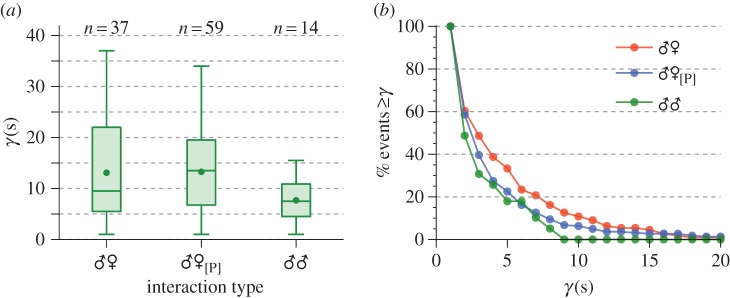
(*a*) Boxplots showing the distribution of per-recording aggregated convergence durations (*γ*(*s*)) for live opposite sex (♂♀), live–playback opposite sex (♂♀_[P]_) and male–male pairs (♂♂). Horizontal lines within each box represent the median, while dots show the mean. The lower and upper edges of the boxes represent, respectively, the lower and upper quartiles of the aggregated convergence times. The boxplot ‘whiskers' give the minimum and maximum of each distribution. Values above each box indicate the number of recordings in which convergence was observed. (*b*) Proportion of convergence events of duration greater than *γ* for unique instances of convergence in the three different recording types.

Having identified unique convergence events within the recordings, statistics of their duration across all paired interaction types can be computed. Figure 8*a* shows the distribution of convergence durations, aggregated on a per-recording basis, for the live opposite sex (♂♀), all opposite sex live–playback (♂♀_[P]_) and same sex (♂♂) pair types. The mean and median convergence time, per recording, are approximately equal for the opposite sex arrangements (both in live and playback form), which also show long tails in their distributions, indicating the presence of long convergence durations in these recordings. Same sex pairs, on the other hand, show both a shorter median and mean convergence duration than opposite sex pairs and a much narrower range (i.e. male–male pairs spend less time in a convergent state, per recording, than male–female ones).

Analysis of individual convergence events reveals a high degree of skew in the data (figure 8*b*), most prominently for the live opposite sex and playback recordings. Most instances of convergence are short, while a small number are notably longer which tends to heavily distort group means. A Kruskal–Wallis test for equality did not indicate a significant effect of pair type on the duration of individual convergence events (*p* > 0.05), although we do observe a decreasing mean (±s.e.) convergence time across live opposite sex, playback and same sex pairs 4.36 ± 0.44 s, 3.53 ± 0.25 s and 2.76 ± 0.37 s for ♂♀, ♂♀_[P]_ and ♂♂, respectively).

**Figure 9.**
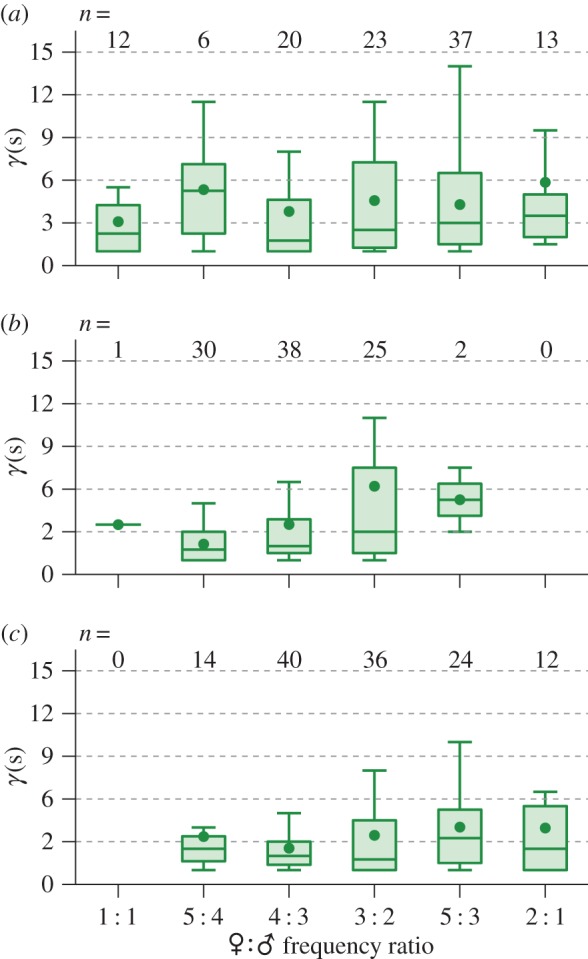
Boxplots of convergence duration (*γ*(*s*)) for unique convergence events at different harmonic combinations in the (*a*) live opposite-sex pair (♂♀) (*b*) live male, playback female (♂♀_P_) and (*c*) live female, playback male (♀♂_P_) cohorts. Numbers at the top of each axis indicate the detected frequency of each harmonic configuration within the recordings. As in figure 8*a*, box lines give (in descending order) the upper quartile, median and lower quartile of the convergence times, and circular markers give the mean.

Our data also invite a more detailed investigation of the different convergence types identified, illustrated schematically in figure 9. The most common harmonic ratios for convergence in live male–female pairs, in decreasing order of occurrence (percentage of recordings in which a convergence type was observed), were 3 : 2 (35%), 5 : 3 (30%), 4 : 3 (16%), 2 : 1 (14%), and 5 : 4 and 1 : 1 (both 8%). Using instead a tally of individual convergence events (as illustrated in figure 9*a*), the ratio 5 : 3 becomes the most frequent (accounting for 33% of unique instances), followed by 3 : 2 (21%), with the remaining order preserved. Convergence at the fundamental (i.e. 1 : 1), typically associated with same sex interactions, was observed only in a single pair of opposite sex mosquitoes (from which repeat recordings were taken).

For males played the sounds of females (♂♀_P_), harmonic ratios of 3 : 2 (42%), 4 : 3 (35%) and 5 : 4 (27%) were detected most frequently in recordings, although in terms of total convergence events 4 : 3 ranked highest (40%), then 5 : 4 (31%) and 3 : 2 (26%). Harmonic convergence at ratios of 5 : 3, 1 : 1, and 2 : 1 was observed twice, once, and not at all, respectively. Conversely, for live females subjected to male playbacks (♀♂_P_), the ratio 4 : 3 was most common both in terms of its prevalence within recordings and as a proportion of the total number of convergence events (correspondingly 36 and 32%), followed closely by 3 : 2 (27 and 29%) and 5 : 3 (24 and 19%). For this dataset, fundamental frequency convergence was not observed.

Across all interaction types, the harmonic ratio at which convergence took place produced no statistically significant difference in the length of the event (Kruskal–Wallis test, *p* > 0.05).

### 4.6. Convergence and flight tone variability

The data we have collected are inherently non-stationary. It is therefore useful to also consider local features when investigating their properties, which may be of behavioural significance at shorter timescales. For example, mosquitoes in pairs have been reported to increase the variability of their wing beat frequencies when compared with those flying alone [31,33]. The resolution of our data allows us to investigate this phenomenon in greater detail, and answer the question of whether the wing beat frequency properties of mosquitoes change as a function of whom their partner is, or how they are interacting with that partner.

To address this, we compute short-time variations in individual mosquitoes' flight tones. We calculate a moving mean and variance (using a rectangular sliding window of length Δ*τ* = 0.25 s) for each wing beat frequency trace, and use these to compute a local coefficient of variation, *c_v_*(*t*) = *σ*(*t*)/*μ*(*t*), i.e. the ratio of the (instantaneous) standard deviation to the (instantaneous) mean. The coefficient of variation, which is a dimensionless quantity, measures spread within a dataset in a standardized way, normalized against the diversity in population means. Since different mosquitoes typically have widely varying mean wing beat frequencies, the coefficient of variation is a more appropriate tool for comparing flight tone dispersion across a population than the standard deviation. As for other measures of spread, a low value of *c*_v_ signifies low variability.

**Figure 10.**
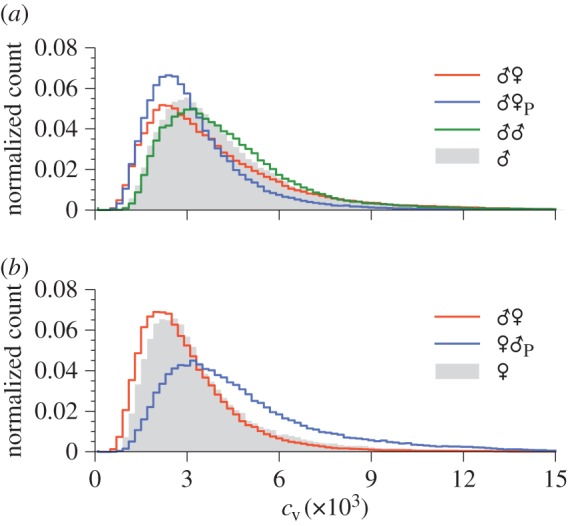
The distribution of coefficients of variation, *c*_v_, for convergent (*a*) male and (*b*) female *A. aegypti* in different pair types. Shaded regions show the corresponding distributions for lone males/females (the ♂ and ♀ datasets).

Figure 10 shows the distribution of local coefficients of variation for convergent male and female mosquitoes in the various recording types. Male *A. aegypti* (figure 10*a*) paired with a female (live or playback) show peaks in their *c*_v_ distributions at lower values than males flying alone, whereas for males paired with another male the converse is true. Female mosquitoes (figure 10*b*) coupled with a live partner also displayed slightly lower dispersion than the solo case. When in the presence of a male playback, however, females show a significantly greater degree of local frequency variation.

The implication here is that when interacting with a female stimulus, male mosquitoes, in general, vary their local flight tones less than when flying alone. The same can be said for females paired with a live male mosquito. Same sex male pairs, however, show a larger amount of local spread. More fundamentally, these results again show the significant behavioural difference between live and playback interaction.

## 5. Conclusion and discussion

In this study, we have provided the first comprehensive, rigorous and quantitative investigation into wing beat frequency interactions between tethered pairs of mosquitoes. To achieve this, we recorded the flight tones of live mosquitoes coupled with various different acoustic stimuli: a live individual of the opposite sex, a pre-recorded playback of the opposite sex and (in the case of male subjects) a live individual of the same sex. In contrast to previous research into auditory communication between mosquitoes [29–31,33], we recorded mosquitoes on separate digital audio channels, for longer time periods (60 s), obtaining data with significantly higher resolution than that has been achieved before. Furthermore, by generating time series of individual frequency components, and subjecting them to a suite of quantitative tools, we were able to precisely define and investigate the phenomenon of harmonic convergence for the first time.

### 5.1. Analysis of the population

Our data suggest that harmonic convergence is a real and active phenomenon, which relies on the ability of both individuals to modulate their wing beat frequencies in response to sounds produced by their partner. There is an alternative, physical, hypothesis to explain the harmonic convergence phenomenon, namely a physical interaction between airborne pressure variations and the mechanical beating of the wing system, which merits some discussion at this point. In another dipteran, the fruit fly *Drosophila melanogaster*, wingbeat is generated myogenically by stretch-activated muscles and hence independently from direct neural input [41]. Their wingbeat has been shown to synchronize to an external stimulus [41]. Could this mechanism also drive rhythmic coupling between mosquito pairs, despite their sound emission profiles being quite different from those of other flies [35]? We cannot, based on our data, rule out this physical interaction hypothesis. However, it has been demonstrated that deafened mosquitoes (both male and female) are incapable of harmonically converging to a playback stimulus tone [29,30]. In addition, deafened males will not attempt to copulate at all with nearby females [22], and flight tone convergence is behaviourally implicated in a given male's mating success [36,37]. These observations point towards a coupling between sensory and locomotive mechanisms in mosquitoes, and that individual control and manipulation of wing beat frequencies, and the observed convergence of flight tones, is a result of active processes. New experiments with tethered mosquitoes, in which one (or both) individual is deafened, could address this issue and further our understanding of mosquito bioacoustics.

We observe a significant difference between the frequency ratio distributions of live mosquito pairs, when compared with those of non-interacting, ‘artificial’ pairs. We generated the latter by combining individual recordings from a database of lone mosquito recordings; this process of ‘randomization’ is readily applicable to other coupled time-series data gathered in behavioural studies.

In the case of live opposite-sex pairs, significant peaks were found at multiple integer ratios, which were not apparent in the control dataset. This indicates that live male and female mosquito pairs do indeed spend a substantial time in a state of frequency convergence, actively maintaining that state. We find significant peaks at previously reported female : male frequency ratios of 3 : 2 and 2 : 1 [30,37], and also at previously unseen ratios of 1 : 1, 5 : 4, 4 : 3 and 5 : 3. These new harmonic ratios do fall within the known acoustic detection range of the mosquito hearing system [47], and there is no reason to discount them.

Male and female mosquitoes played back the sounds of conspecifics also displayed convergence at the same fundamental ratios as the live duets, although at different intensities. The ratio distribution of males listening to pre-recorded females is somewhat similar to that of the ‘artificial’ pair data. By contrast, data from live females paired with male playback recordings indicates considerable convergence to the playback stimulus. This provides further evidence of active female participation in frequency convergence events, and of differences between male and female responses to auditory stimuli.

When paired with a member of the same sex, male mosquitoes have been demonstrated to frequency avoid [29,31,32], but the data reported here reveal that their interaction is more complicated than this. Our analysis of fundamental wing beat frequency distribution between males indicates a mix of convergence and avoidance. Further investigation of this phenomenon, which does not feature in the analysis of combined lone males, indicates that live male–male pairs occupy two different states in the frequency domain: males that converge spend much of their time at a wing beat frequency less than 25 Hz apart, whereas the separation for pairs that do not converge tends to be over 50 Hz.

### 5.2. Analysis of individual convergence events

Frequency convergence was a ubiquitous feature of acoustic interactions between mosquito pairs in all experimental arrangements tested. We observed at least one convergence event in 86% of recordings of live opposite sex pairs, and in 87% of playback tests. This is markedly higher than the convergence rate of 67% reported for *A. aegypti* in [30], probably because our analysis considers a wider range of harmonic ratios for potential convergence. In live pairs, convergence was most commonly observed in recordings at a female : male harmonic combination of 3 : 2, followed closely by 5 : 3. Convergence at other ratios (1 : 1, 5 : 4, 4 : 3 and 2 : 1) was observed, but was less pronounced. For playback recordings, overall, 4 : 3 was marginally more widespread than 3 : 2, yet both ratios stood out from other convergence types.

For male–male pairs, the convergence rate was 47%: convergence takes place less frequently between same sex pairs than opposite sex. However, across each of the three treatment groups, where convergence was detected within a given recording, it tended to be repeated at a similar rate. That is, in convergent datasets, multiple instances were found in over 70% of all pair recordings, whether same or opposite sex, live or playback. Thus, when a convergent frequency interaction takes place between males, it is just as likely to be repeated as in a male–female pair. Again, this does not indicate a strictly avoidant response of males to one another.

There is no statistically significant effect of interaction type (live opposite/same sex pair or opposite sex playback) or harmonic frequency combination on the duration of individual convergence events. The majority of convergence events in all pair types are short (less than or equal to 1.5 s). In opposite sex pairs, a greater proportion are lengthier; this could be symptomatic of ‘hunting’ behaviours described in same sex pairs [29], whereby males quickly modulate their wing beat frequencies between transient bouts of convergence.

### 5.3. Implications for future research

It is pertinent to consider mosquito flight tone behaviours in the broader context of their ecology. It has been shown that the ability of a male to match his flight frequency to that of a female is a genetically inheritable trait [37], and may be used by females to assess male fecundity [36]. Furthermore, the presence of harmonic convergence between a pair was found to be positively correlated with successful copulation, and that females were less likely to reject males who demonstrated an ability to converge [37]. Convergence may also confer sub-species recognition and membership in mixed-form populations of *An. gambiae* [33]. It is unclear, however, exactly what factors lead to or initiate convergence. The nature of the information contained within the convergent signal remains elusive. For instance, do different convergence types, or lengths, produce different success rates? Or does the ‘accuracy’ of the convergence event transmit information between the individuals about their quality in the context of sexual selection? Even basic consequences of a change in the wing beat frequency of a mosquito, such as whether there is an alteration of speed or direction of flight, are poorly understood; such questions must be addressed before the collective behaviour of groups can be fully comprehended.

The data suggest that, during convergence, pairs of males more accurately match their flight frequencies than pairs of the opposite sex. But why do males converge at all? Male mosquitoes are stimulated by a range of sound frequencies (rather than a fixed pitch), and any tone within that range could elicit some sort of copulatory response [22]. In a natural swarm environment, auditory interactions are time-limited by the spatio-temporal aspects of free flight. As acoustic signalling plays a crucial role in mating behaviour, rapid gender identification is important to reduce the number of wasted pursuits. It may be that males use such dynamic frequency cues to inform their decision-making within the collective swarm aggregation. Indeed, such a notion has recently been proposed in another swarming insect species: midges [48].

Understanding the responses of one male to his neighbours is critical if we are to gain insights into the dynamics of swarming in mosquitoes. Our analysis presents some novel possibilities as to what occurs when males interact with members of the same and opposite sex, and generates many new research questions concerning mosquito bioacoustics. Clearly, the informational content of the convergent signal remains unknown, be it for pairs or more complex associations within a swarm. Only by pursuing such avenues, and applying rigorous analytical tools, can we begin to fully appreciate the role of sound in mosquito ethology.

## Data accessibility

Mosquito auditory interaction data: University of Bristol data.bris Research Data Repository (http://dx.doi.org/10.5523/bris.1a44saul2ijj31u0raipvlims6).

## References

[1] Butail S, Manoukis N, Diallo M, Ribeiro JM, Lehmann T, Paley DA 2012 Reconstructing the flight kinematics of swarming and mating in wild mosquitoes. J. R. Soc. Interface 9, 2624–2638. (10.1098/rsif.2012.0150) 22628212 PMC3427502

[2] Butail S, Manoukis NC, Diallo M, Ribeiro JMC, Paley DA 2013 The dance of male *Anopheles gambiae* in wild mating swarms. J. Med. Entomol. 50, 552–559. (10.1603/ME12251) 23802449 PMC4780853

[3] Kelley DH, Ouellette NT 2013 Emergent dynamics of laboratory insect swarms. Sci. Rep. 3, 1073 (10.1038/srep01073) 23323215 PMC3545223

[4] Shishika D, Manoukis NC, Butail S, Paley D 2014 Male motion coordination in anopheline mating swarms. Sci. Rep. 4, 6318 (10.1038/srep06318) 25212874 PMC4161964

[5] Puckett JG, Ouellette NT 2014 Determining asymptotically large population sizes in insect swarms. J. R. Soc. Interface 11, 20140710 (10.1098/rsif.2014.0710) 25121646 PMC4233756

[6] Puckett JG, Kelley DH, Ouellette NT 2014 Searching for effective forces in laboratory insect swarms. Sci. Rep. 4, 4766 (10.1038/srep04766) 24755944 PMC3996478

[7] Ballerini M et al. 2008 Empirical investigation of starling flocks: a benchmark study in collective animal behaviour. Anim. Behav. 76, 210–215. (10.1016/j.anbehav.2008.02.004)

[8] Bialek W, Cavagna A, Giardina I, Mora T, Silvestri E, Viale M, Walczak AM 2012 Statistical mechanics for natural flocks of birds. Proc. Natl Acad. Sci. USA 109, 4786–4791. (10.1073/pnas.1118633109) 22427355 PMC3324025

[9] Lopez U, Gautrais J, Couzin ID, Theraulaz G 2012 From behavioural analyses to models of collective motion in fish schools. Interface Focus 2, 693–707. (10.1098/rsfs.2012.0033) 24312723 PMC3499128

[10] Sumpter DJT 2006 The principles of collective animal behaviour. Phil. Trans. R. Soc. Lond. B 361, 5–22. (10.1098/rstb.2005.1733) 16553306 PMC1626537

[11] Buhl J, Sumpter DJT, Couzin ID, Hale JJ, Despland E, Miller ER, Simpson SJ 2006 From disorder to order in marching locusts. Science 312, 1402–1406. (10.1126/science.1125142) 16741126

[12] Charlwood JD, Jones MDR 1980 Mating in the mosquito, *Anopheles gambiae* s.l.: II. Swarming behaviour. Physiol. Entomol. 5, 315–320. (10.1111/j.1365-3032.1980.tb00241.x)

[13] Yuval B, Bouskila A 1993 Temporal dynamics of mating and predation in mosquito swarms. Oecologia 95, 65–69. (10.1007/BF00649508) 28313313

[14] Clements AN 1999 The biology of mosquitoes, *vol. 2*: sensory reception and behaviour, ch. Mating, pp. 378–380. London, UK: Chapman & Hall.

[15] Takken W, Costantini C, Dolo G, Hassanali A, Sagnon NF, Osir E 2006 Mosquito mating behaviour. In Bridging laboratory and field research for genetic control of disease vectors, vol. 11 *of Wageningen UR Front is Series* (eds Knols BGJ, Louis C), pp. 183–188. Berlin, Germany: Springer.

[16] Manoukis NC, Diabate A, Abdoulaye A, Diallo M, Yaro AS, Ribeiro JMC, Lehmann T 2009 Structure and dynamics of male swarms of *Anopheles gambiae*. J. Med. Entomol. 46, 227–235. (10.1603/033.046.0207) 19351073 PMC2680012

[17] Robert D 2009 Insect bioacoustics: mosquitoes make an effort to listen to each other. Curr. Biol. 19, R446–R449. (10.1016/j.cub.2009.04.021) 19515350

[18] Diabaté A, Yaro AS, Dao A, Diallo M, Huestis DL, Lehmann T 2011 Spatial distribution and male mating success of *Anopheles gambiae* swarms. BMC Evol. Biol. 11, 184–196. (10.1186/1471-2148-11-184) 21711542 PMC3146442

[19] Oliva CF, Damiens D, Benedict MQ 2013 Male reproductive biology of *Aedes* mosquitoes. Acta Tropica 132 S12–S19. (10.1016/j.actatropica.2013.11.021) 24308996

[20] Cabrera M, Jaffe K 2007 An aggregation pheromone modulates lekking behavior in the vector mosquito *Aedes aegypti* (Diptera: Culicidae). J. Am. Mosquito Control Assoc. 23, 1–10. (10.2987/8756-971X(2007)23%5B1:AAPMLB%5D2.0.CO;2) 17536361

[21] Johnston C 1855 Original communications: auditory apparatus of the *Culex* mosquito. Q. J. Microsc. Sci. 1, 97–102.

[22] Roth LM 1948 A study of mosquito behavior. An experimental laboratory study of the sexual behavior of *Aedes aegypti* (Linnaeus). Am. Midland Nat. 40, 265–352. (10.2307/2421604)

[23] Wishart G, Riordan DF 1959 Flight responses to various sounds by adult males of *Aedes aegypti*. Can. Entomol. 91, 181–191. (10.4039/Ent91181-3)

[24] Belton P 1974 An analysis of direction finding in male mosquitoes. In Experimental analysis of insect behaviour (ed. Knowles LB), pp. 139–148. Berlin, Germany: Springer.

[25] Charlwood JD, Pinto J, Sousa CA, Madsen H, Ferreira C, Do Rosario VE 2002 The swarming and mating behaviour of *Anopheles gambiae* s.s. (Diptera: Culicidae) from São Tomé Island. J. Vect. Ecol. 27, 178–183. 12546454

[26] Göpfert MC, Robert D 2001 Active auditory mechanics in mosquitoes. Proc. R. Soc. Lond. B 268, 333–339. (10.1098/rspb.2000.1376) PMC1088611 11270428

[27] Jackson JC, Robert D 2006 Nonlinear auditory mechanism enhances female sounds for male mosquitoes. Proc. Natl Acad. Sci. USA 103, 16 734–16 739. (10.1073/pnas.0606319103) PMC1636524 17068125

[28] Avitabile D, Homer M, Champneys AR, Jackson JC, Robert D 2010 Mathematical modelling of the active hearing process in mosquitoes. J. R. Soc. Interface 7, 105–122. (10.1098/rsif.2009.0091) 19447819 PMC2839377

[29] Gibson G, Russell I 2006 Flying in tune: sexual recognition in mosquitoes. Curr. Biol. 16, 1311–1316. (10.1016/j.cub.2006.05.053) 16824918

[30] Cator LJ, Arthur BJ, Harrington LC, Hoy RR 2009 Harmonic convergence in the love songs of the dengue vector mosquito. Science 323, 1077–1079. (10.1126/science.1166541) 19131593 PMC2847473

[31] Warren B, Gibson G, Russell IJ 2009 Sex recognition through midflight mating duets in *Culex* mosquitoes is mediated by acoustic distortion. Curr. Biol. 19, 485–491. (10.1016/j.cub.2009.01.059) 19269180

[32] Gibson G, Warren B, Russell IJ 2010 Humming in tune: sex and species recognition by mosquitoes on the wing. J. Assoc. Res. Otolaryngol. 11, 527–540. (10.1007/s10162-010-0243-2) 20976515 PMC2975882

[33] Pennetier C, Warren B, Dabiré KR, Russell IJ, Gibson G 2010 ‘Singing on the wing’ as a mechanism for species recognition in the malarial mosquito *Anopheles gambiae*. Curr. Biol. 20, 131–136. (10.1016/j.cub.2009.11.040) 20045329

[34] Aldersley A, Champneys AR, Homer M, Robert D 2014 Frequency analysis of mosquito flight tones. J. Acoust. Soc. Am. 136, 1982–1989. (10.1121/1.4895689) 25324097

[35] Arthur BJ, Emr KS, Wyttenbach RA, Hoy RR 2014 Mosquito (*Aedes aegypti*) flight tones: frequency, harmonicity, spherical spreading, and phase relationships. J. Acoust. Soc. Am. 135, 933–941. (10.1121/1.4861233) 25234901 PMC3985972

[36] Cator LJ, Ng'Habi KR, Hoy RR, Harrington LC 2010 Sizing up a mate: variation in production and response to acoustic signals in *Anopheles gambiae*. Behav. Ecol. 21, 1033–1039. (10.1093/beheco/arq087)

[37] Cator LJ, Harrington LC 2011 The harmonic convergence of fathers predicts the mating success of sons in *Aedes aegypti*. Anim. Behav. 82, 627–633. (10.1016/j.anbehav.2011.07.013) 22003255 PMC3190198

[38] de Mello Vigoder F, Ritchie MG, Gibson G, Peixoto AA 2013 Acoustic communication in insect disease vectors. Meḿorias do Instituto Oswaldo Cruz, 108, 26–33. (10.1590/0074-0276130390) 24473800 PMC4109177

[39] Arthur BJ, Sunayama-Morita T, Coen P, Murthy M, Stern DL 2013 Multi-channel acoustic recording and automated analysis of *Drosophila* courtship songs. BMC Biol. 11, 11 (10.1186/1741-7007-11-11) 23369160 PMC3599446

[40] Dudley R 2000 In The biomechanics of insect flight: form, function, evolution. Princeton, NJ: Princeton University Press.

[41] Bartussek J, Kadir Mutlu A, Zapotocky M, Fry SN 2013 Limit-cycle-based control of the myogenic wingbeat rhythm in the fruit fly *Drosophila*. J. R. Soc. Interface 10, 20121013 (10.1098/rsif.2012.1013) 23282849 PMC3565748

[42] Cator LJ, Arthur BJ, Ponlawat A, Harrington LC 2011 Behavioral observations and sound recordings of free-flight mating swarms of *Ae. aegypti* (Diptera: Culicidae) in Thailand. J. Med. Entomol. 48, 941–946. (10.1603/ME11019) 21845959 PMC4948640

[43] Huang NE, Wu S, Long SR, Arnold KC, Chen X, Blank K 2009 On instantaneous frequency. Adv. Adaptive Data Anal. 1, 177–229. (10.1142/S1793536909000096)

[44] Keogh FJ, Pazzani MJ 2000 Scaling up dynamic time warping for datamining applications. In *Proc. of the sixth ACM SIGKDD Int. Conf. on Knowledge discovery and data mining*, *Boston, Massachusetts*, pp. 285–289. New York, NY: ACM.

[45] Keogh EJ, Chakrabarti K, Pazzani MJ, Mehrotra S 2001 Dimensionality reduction for fast similarity search in large time series databases. Knowl. Inform. Syst. 3, 263–286. (10.1007/PL00011669)

[46] Göpfert MC, Briegel H, Robert D 1999 Mosquito hearing: sound-induced antennal vibrations in male and female *Aedes aegypti*. J. Exp. Biol. 202, 2727–2738. 10504309 10.1242/jeb.202.20.2727

[47] Arthur BJ, Wyttenbach RA, Harrington LC, Hoy RR 2010 Neural responses to one- and two-tone stimuli in the hearing organ of the dengue vector mosquito. J. Exp. Biol. 213, 1376–1385. (10.1242/jeb.033357) 20348350 PMC3183485

[48] Puckett JG, Ni R, Ouellette NT 2015 Time–frequency analysis reveals pairwise interactions in insect swarms. Phys. Rev. Lett. 114, 258103 (10.1103/PhysRevLett.114.258103) 26197145

